# Marine-Derived Stichloroside C2 Inhibits Epithelial–Mesenchymal Transition and Induces Apoptosis through the Mitogen-Activated Protein Kinase Signalling Pathway in Triple-Negative Breast Cancer Cells

**DOI:** 10.1155/2022/6449984

**Published:** 2022-05-14

**Authors:** Chuang Cui, Chen-Huan Ding, Fang-Fang Liu, Jing-Rong Lu, Shi-Yun Zheng, Hou-Wen Lin, Wei-Kang Zhu, Fan Yang, He Li

**Affiliations:** ^1^Shanghai Municipal Hospital of Traditional Chinese Medicine, Shanghai University of Traditional Chinese Medicine, Shanghai 200071, China; ^2^Research Center for Marine Drugs, State Key Laboratory of Oncogenes and Related Genes, Department of Pharmacy, Renji Hospital, School of Medicine, Shanghai Jiao Tong University, Shanghai 200127, China; ^3^Traditional Chinese Medicine Department, Renji Hospital, School of Medicine, Shanghai Jiao Tong University, Shanghai 200127, China; ^4^Shanghai University of Traditional Chinese Medicine, Shanghai 201203, China

## Abstract

**Background:**

Triterpenoid saponins from sea cucumbers exhibit significant antitumour, antifungal, and antibacterial activities. However, the associated molecular mechanisms have yet to be elucidated. In this study, we screened and explored the antitumour activity and underlying mechanisms of triterpenoid saponins isolated from *Thelenota ananas*.

**Methods:**

We isolated and purified sea cucumber saponins, determined their chemical structures, and confirmed their function *in vitro*. We also screened and explored the antitumour activity and underlying mechanisms of triterpenoid saponins isolated from *Thelenota ananas*.

**Results:**

Four saponins were discovered from sea cucumber *Thelenota ananas* collected from the South China Sea. We found that stichloroside C2 (STC2) inhibited the proliferation and clonogenesis of the human triple-negative breast cancer (TNBC) cell line MDA-MB-231 and mouse TNBC cell line 4 T1 in a dose-dependent manner and induced apoptosis and cycle arrest in these two TNBC cell lines. STC2 induced DNA damage in two TNBC cell lines and significantly increased the protein expression level of the DNA double-strand break marker *γ*-H2AX. STC2 downregulated the protein expression levels of phosphorylated cyclin-dependent kinase 1 (CDK1), cyclin B1, CDK2, and cyclin A2 in MDA-MB-231 and 4 T1 cells. STC2 upregulated Bax and cleaved PARP protein expression in two types of breast cancer cells. In addition, STC2 promoted E-cadherin expression; inhibited vimentin expression; upregulated the phosphorylation levels of the mitogen-activated protein kinase (MAPK) signalling pathway-related proteins p38, JNK, and ERK1/2; and downregulated Akt phosphorylation.

**Conclusions:**

STC2 exerts anti-TNBC activity, inhibits epithelial–mesenchymal transition (EMT), and induces apoptosis by regulating the cell cycle, EMT-related proteins, and MAPK signalling pathway.

## 1. Introduction

Breast cancer is the most common malignancy in women in China. It has replaced lung cancer as the world's most common cancer [1, 2] and is one of the most life-threatening diseases affecting women. Triple-negative breast cancer (TNBC) is a special type of breast cancer, accounting for approximately 10–20.8% of total breast cancer cases. Compared to other types of breast cancer, TNBC has apparent differences in its biological behaviour and clinicopathological examination results, and its clinical prognosis is often worse than that of other types of breast cancer.

TNBC is highly invasive and metastatic and is closely related to the occurrence of epithelial–mesenchymal transition (EMT) [3]. EMT was first discovered during embryonic development, and the development of mesenchymal cells promotes cell invasion and proliferation [4]. When adult epidermal cells are damaged, the corresponding EMT phenomena also occur. During the occurrence and development of tumour cells, EMT can also cause tumour cells to lose some of the characteristics of epithelial cells and acquire some characteristics of mesenchymal cells, thereby enhancing the detachment and invasiveness of tumour cells. During EMT, epithelial cells lose polarity, the contact between surrounding cells and stromal cells is reduced, intercellular interactions are reduced, and cell migration and motility are enhanced. Simultaneously, cell phenotypes, such as keratin filaments and E-cadherin, are altered, and epithelial phenotypes are lost. Decreased E-cadherin levels lead to decreased cell adhesion, invasion, and metastasis. The loss of E-cadherin expression is considered the most significant feature of EMT [5]. At the same time, interstitial phenotypes, such as vimentin, N-cadherin, and other expressions, were increased. The mitogen-activated protein kinase (MAPK) signalling pathway is a common pathway of extracellular signal stimulation leading to a nuclear response and is an intracellular signalling molecule-mediating EMT [6]. Many signalling molecules can mediate EMT through the MAPK signalling pathway [7, 8].

Standardised treatment options for breast cancer in clinical practice include surgery, chemotherapy, radiotherapy, and targeted therapy; however, these treatments are often accompanied by side effects [9]. Furthermore, owing to the particularity of its occurrence site, it often causes great physical and mental distress to patients. Studies have shown that bioactive natural products usually have fewer toxic side effects than conventional therapies in the treatment of breast cancer [10].

Marine natural products derived from various marine organisms have received increasing attention from researchers in the field of life science as novel sources of drugs for various human diseases, including a variety of tumours. The sea cucumber, a marine echinoderm, is not only considered a delicacy in China, but has also been recorded as a valuable medicinal organism. The main metabolites of sea cucumbers include saponins, polysaccharides, and peptides, of which triterpenoid saponins are the most important secondary metabolites. The triterpenoid saponins of sea cucumbers are unique to them and possess biological activities associated with membrane permeability, such as haemolysis, cytotoxicity, and antifungal activity [11]. *Thelenota ananas* is a popular edible sea cucumber distributed throughout China's Hainan, Xisha, Zhongsha, Dongsha, and Nansha Islands. In this paper, the chemical isolation and pharmacological studies of the sea cucumber saponins are reported.

## 2. Materials and Methods

### 2.1. Samples and Reagents

#### 2.1.1. Isolation of Sea Cucumber Saponins

*Thelenota ananas* samples were collected in the vicinity of Yongxing Island, one of the Xisha Islands in the South China Sea. The samples were stored at -20°C in the Research Center for Marine Drugs, Renji Hospital. The sample (1, 500 g, dry weight) was extracted with 75% EtOH/H_2_O to obtain a crude extract (11.7 g). The total extract was subjected to silica gel column chromatography using stepwise dichloromethane/methanol (15 : 1, 8 : 1, 6 : 1, pure methanol, *v*/*v*) to obtain four fractions (HSA1–HSA4). Subfraction HSA1 (3.52 g) was subjected to medium-pressure liquid chromatography (MPLC) with an octadecyl silane (ODS) column eluted with a gradient of 10% to 100% (*v*/*v*) MeOH/H_2_O to give A1029–A1034. A1033 (291 mg) was isolated by semi-preparative reversed-phase HPLC (5 *μ*m; YMC, Kyoto, Japan) and eluted with 44% MeCN/H_2_O (0.1% HCOOH) to yield compound **1** (23 mg, tR = 27 min, 2.0 mL/min), compound **2** (12 mg, tR = 29 min, 2.0 mL/min), compound **3** (60 mg, tR = 41 min, 2.0 mL/min), and compound **4** (11 mg, tR = 14 min, 2.0 mL/min).

#### 2.1.2. Other Reagent Sources

Cell Counting Kit-8 (CCK-8; CK04–50000 T; Dojindo, Kumamoto, Japan), Annexin V-FITC/propidium iodide (PI) kit (AD10-50 T; Dojindo, Japan), dimethyl sulfoxide (DMSO; 1129E033; Solarbio, Beijing, China), foetal bovine serum (FBS; 10,099; Gibco, Waltham, MA, USA), trypsin-EDTA solution (25,200,072; Gibco), Dulbecco's modified Eagle medium (DMEM; 11054001, Gibco), RPMI 1640 medium (A1049101; Gibco), Leibovitz's L15 medium (11415064; Gibco), rapamycin (S1039; Selleckchem, Houston, TX, USA), 2.5% glutaraldehyde fixation (DF0156, Leagene Biological, Beijing, China), and bicinchoninic acid (BCA) protein assay kit (Beyotime Institute of Biotechnology, Shanghai, China) were used. The *β*-actin (13E5), cyclin B1 (D5C10), cyclin A2 (E6D1J), phospho-cdc2 (Tyr15) (10A11), cyclin-dependent kinase 2 (CDK2; E8J9T), phospho-histone H2A.X (Ser139) (D7T2V), GAPDH (D16H11), Bax (2D2), cleaved PARP (Asp214) (D64E10), vimentin (D21H3), E-cadherin (4A2), p38 MAPK (D13E1), phospho-p38 MAPK (Thr180/Tyr182) (D3F9), SAPK/JNK antibody, phospho-SAPK/JNK (Thr183/Tyr185) (G9), Akt (pan) (C67E7), Phospho-Akt (Ser473) (D9E), p44/42 MAPK (Erk1/2) (137F5), and phospho-p44/42 MAPK (Erk1/2) (Thr202/Tyr204) (D13.14.4E) were all purchased from Cell Signalling Technology (CST; Danvers, MA, USA).

### 2.2. Cell Culture

Breast cancer cell lines (MDA-MB-231, MCF-7, and 4 T1) and the normal ovarian epithelial cell line (IOSE-80) were obtained from the Chinese Academy of Sciences Stem Cell Bank. The cell lines were treated with 10% FBS, 1% L-glutamine, 1% penicillin–streptomycin mixture, L-15, R-PMI-1640, and DMEM in a humid incubator at 37°C.

### 2.3. Cell Viability Was Detected by the CCK-8 Method

The water-soluble chemicals in the Cell Counting Kit-8 can be reduced to orange or yellow dyes using electron carriers; these dyes can be dissolved in the culture medium. The amount of dye produced by this reaction is proportional to the number of living cells. As the colour difference can be distinguished by the naked eye, the number of living cells in the cell proliferation or toxicity experiments can be determined. The cells to be tested in the stable growth stage were digested with an appropriate amount of 0.25% trypsin, blown into a single-cell suspension state, blown, mixed evenly, and counted into a 5 × 10^5^ cells/mL single-cell suspension, which was added to the prepared 96-well plate. Each group comprised three wells, and the number of cells in each well was approximately 5,000. A blank control group was established after the cells were cultured overnight in an incubator and affixed to the wall. In the experimental group, the well was added with the related compounds to be tested with the same dilution in the same proportion and cultured in an incubator at 37°C for 24 h. Subsequently, 10 *μ*L of CCK-8 solution was added to each well, and the culture plate was incubated for an appropriate time according to different cells. The multifunctional microplate detector was set to 450 nm, and the absorbance values of all holes were measured.

### 2.4. Cell Clonal Formation Experiment

Logarithmic growth cells were inoculated into 6-well plates with three wells for each group and no more than 1,000 cells in each well. The cells were incubated overnight in a 37°C constant temperature incubator. Sea cucumber saponins were added at concentrations of 0.25, 0.5, and 1 *μ*M, and a blank control group was used. After culturing at 37°C for 24 h, replace the fresh medium; the 6-well plates were taken out, washed with phosphate-buffered saline (PBS), and the cells were fixed with 4% paraformaldehyde at room temperature (ca. 20°C) for more than 10 min. The cells were then stained with crystal violet solution to avoid light for more than 15 min and counted.

### 2.5. Cell Cycle and Apoptosis Analysis

The cell cycle and apoptosis were analysed by flow cytometry. The DNA content of cells varies between cycles, and propidium iodide (PI) can stain the DNA of cells and be detected using flow cytometer filters. Therefore, this reagent can be used for cell cycle analysis using flow cytometry. Stably growing cells were digested with trypsin and then beaten into a single-cell suspension. The cells were inoculated in 6-well plates at a concentration of 1 × 10^6^/well and incubated at 37°C overnight in an incubator. The cells were collected into test tubes after 24 h of treatment with a series of different concentrations of sea cucumber saponins. Subsequently, 700 *μ*L of anhydrous ethanol and 300 *μ*L of PBS (both precooled in a 4°C refrigerator) were added and the cells were placed in a -20°C freezer and left overnight. The cells were centrifuged at 1,200 rpm for 5 min, the supernatant was discarded, and 1 mL of PBS (precooled to 4°C) was added to wash the resuspended cells. The cells were centrifuged at 1,200 rpm for 5 min, after which the supernatant was discarded, 500 *μ*L PI/RNAse reagent was added to each tube, and the cells were incubated for 15 min at room temperature without light. Flow cytometry was used to detect the viruses.

Annexin V staining can be used to detect cell membrane changes at the early stage of apoptosis, while PI can be used to distinguish late apoptotic cells from dead and necrotic cells. A combination of Annexin V and PI staining was used to detect cell apoptosis at all stages.

### 2.6. Western Blotting

The total proteins from treated cells and tissues were extracted using radio-immunoprecipitation assay (RIPA) buffer containing 1 mmol/L phenylmethylsulphonyl fluoride and quantified using a BCA protein analysis kit. Protein bands were separated by sodium dodecyl–sulphate polyacrylamide gel electrophoresis and transferred to a polyvinylidene fluoride (PVDF) membrane. After PBS with Tween 20 was sealed with 5% bovine serum albumin in phosphate buffer for 1 h, the PVDF membrane was incubated overnight with primary antibody at 4°C. After washing the membrane three times, the secondary antibody coupled with horseradish peroxidase was incubated at room temperature for 1 h. Finally, western blotting was visualised using an enhanced chemiluminescence kit (Beyotime Institute of Biotechnology, Shanghai, China) according to the manufacturer's instructions. The intensity of the western blotting bands was determined using ImageJ software (version 1.46; National Institutes of Health [NIH], Bethesda, MD, USA).

### 2.7. Statistical Analysis

All data in the experiment are expressed as the mean ± SEM, and GraphPad Prism software (version 8.0) was used to draw graphs and perform statistical analysis. One-way analysis of variance was used to compare data between multiple groups involved in the experiment, and Dunnett's *t*-test was used for relevant experimental data requiring pairwise comparison between groups. All the data processing involved in the experiment were obtained from the original experimental data. Differences in the experimental data were considered statistically significant at *P* < 0.05.

## 3. Results

### 3.1. Structural Identification of Four Sea Cucumber Saponins

**Figure 1:**
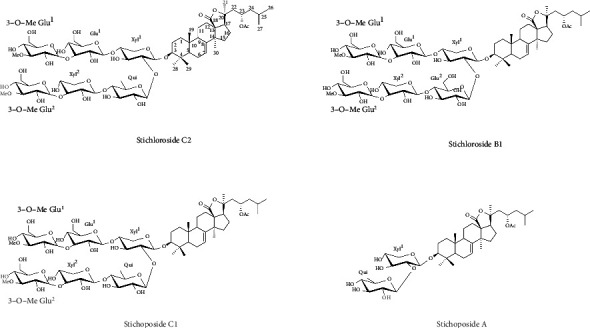
Structures of the four detected sea cucumber saponins.

Four sea cucumber saponin compounds were obtained from the extraction of *Thelenota ananas*. Using a combination of high-resolution electrospray ionisation mass spectrometry, extensive nuclear magnetic resonance spectroscopic analyses, and a review of the existing literature (Supplementary Materials, Table S1, Table S2), four compounds and their structures were identified: compound **1** stichloroside C2 (STC2), compound **2** stichloroside B1 (STB1) [12, 13], compound **3** stichoposide C1 (STC1) [14], and compound **4** stichoposide A (STA) [15, 16] (Figure 1). After preliminary activity detection (Supplementary Materials, Figure S1, Table S3), we decided to select STC2 with strong antitumour activity and low cytotoxicity to normal cells for subsequent pharmacological studies.

### 3.2. STC2 Inhibits the Proliferation of Breast Cancer Cells

**Figure 2:**
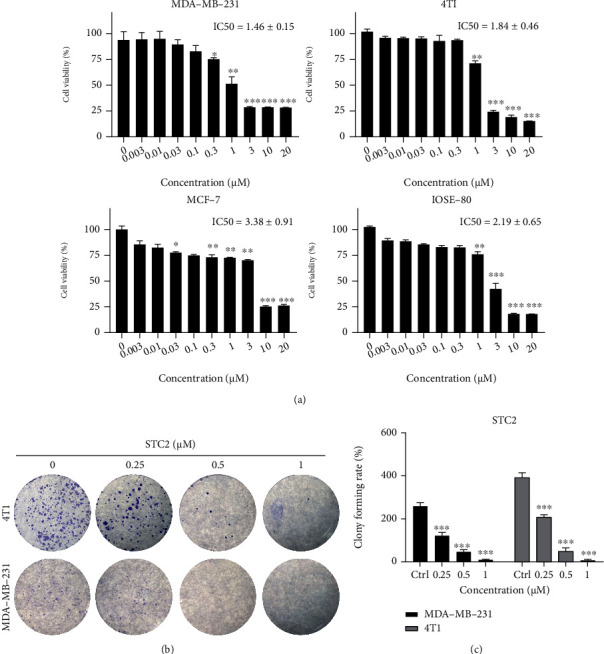
Stichloroside C2 (STC2) can inhibit the proliferation of breast cancer cells. (a) Three breast cancer cell lines, comprising a human breast cancer cell line (MCF-7), human triple-negative breast cancer (TNBC) cell line (MDA-MB-231), and mouse TNBC cell line (4 T1), and a normal epithelial cell line (IOSE-80) were selected and treated with a series of different concentrations of STC2 after treatment for 24 h, the cell viability was detected using the Cell Counting Kit-8 (CCK-8) method. ∗*P* < 0.05, ∗∗*P* < 0.01, ∗∗∗*P* < 0.001, compared to the control group. (b, c) Inhibition of TNBC cell clone formation by sea cucumber saponin monomer. Human MDA-MB-231 cells and mouse 4 T1 cells were treated with STC2 at different concentrations (0.25, 0.5, and 1 *μ*M) for 24 h. The cells were cultured for 1–2 weeks and stained with crystal violet. The number of clones formed was detected. ∗∗*P* < 0.01, ∗∗∗*P* < 0.001, compared to the control group.

To detect the antitumour activity of sea cucumber saponins, three breast cancer cell lines, comprising a human breast cancer cell line (MCF-7), human TNBC cell line (MDA-MB-231), and mouse TNBC cell line (4 T1), and a normal epithelial cell line (IOSE-80), were selected and treated with a series of different concentrations of STC2 (0.03, 0.1, 0.3, 1, 3, 10, and 20 *μ*M) for 24 h. The viability of cells was determined using the CCK-8 method. The results showed that STC2 significantly inhibited the activity of the three kinds of breast cancer cells, with IC_50_ values below 5 *μ*M, indicating significant antitumour activity (Figure 2(a)). However, cucumber saponin also showed varying degrees of lethality against the IOSE-80 cells, which may be related to its widely reported haemolytic activity.

To verify the inhibitory effect of STC2 on the clonal formation of breast cancer cells, a series of STC2 with different concentrations (0.25 *μ*M, 0.5, and 1 *μ*M) were used to treat human MDA-MB-231 cells and mouse 4 T1 cells for 24 h. After 1–2 weeks of culture, the cells were stained with crystal violet to determine the number of clones. The results showed that 0.25 *μ*M, 0.5 *μ*M, and 1 *μ*M STC2 reduced the number of MDA-MB-231 cell clones by 52.44 ± 4.56% (*P* < 0.001), 81.43 ± 3.83% (*P* < 0.001), and 95.90 ± 3.41% (*P* < 0.001) (Figure 2(b)); and the number of 4 T1 cell clones by 46.92 ± 3.32% (*P* < 0.001), 87.32 ± 3.74% (*P* < 0.001), and 97.64 ± 2.99% (*P* < 0.001), respectively, compared to those in the control group. In conclusion, STC2 had a significant inhibitory effect on the clonal formation of the two types of breast cancer cells.

### 3.3. STC2 Induces TNBC Cell Cycle Arrest

**Figure 3:**
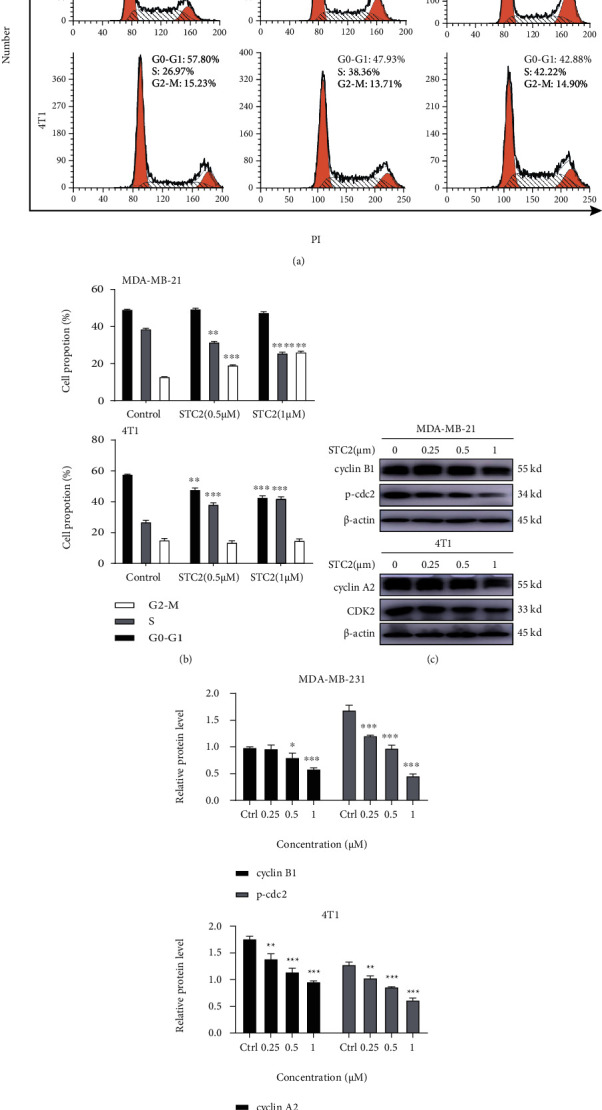
Cell cycle arrest of triple-negative breast cancer (TNBC) induced by sea cucumber saponins. (a) The human TNBC cell line MDA-MB-231 and the mouse TNBC cell line 4 T1 were treated with 0.5 and 1 *μ*M of stichloroside C2 (STC2) for 24 h. The cells were digested and collected, and the cycle changes of the two cells were detected by flow cytometry with propidium iodide (PI). (b) Prism 8.0 was used for statistical analysis of the experimental data, which were expressed as the mean ± SEM of three independent experiments. ∗∗∗*P* < 0.001, compared to the control group. (c) MDA-MB-231 and 4 T1 cells were treated with different concentrations of STC2 (0.25, 0.5, and 1 *μ*M) for 6 h, and the expression of cycle-related proteins in MDA-MB-231 and 4 T1 cells was detected by western blot. (d) Data are presented as the mean ± SEM of three independent experiments, ∗*P* < 0.05, ∗∗*P* < 0.01, ∗∗∗*P* < 0.001, compared to the control group.

To test whether the inhibitory effect of STC2 on the proliferation of TNBC cells is related to the cell cycle, MDA-MB-231 and 4 T1 were treated with STC2 at different concentrations (0.5 and 1 *μ*M) for 24 h. The cycle changes in the two cell types were detected by flow cytometry after digestion and collection. The results showed (Figures 3(a) and 3(b)) that in MDA-MB-231 cells treated with STC2, the number of cells in the G2/M phase increased (*P* < 0.001), while the number of cells in the G0/G1 and S phases remained unchanged or decreased. Therefore, it can be concluded that the cell cycle of MDA-MB-231 cells treated with STC2 was arrested in the G2/M phase. In 4 T1 cells treated with STC2, the number of cells in the S phase increased (*P* < 0.001), whereas the number of cells in the G0/G1 and G2/M phases remained unchanged or decreased. This indicated that the cycle of 4 T1 cells treated with STC2 was blocked in the S phase. These results suggest that STC2 may inhibit the proliferation of breast cancer cells by inducing cell cycle arrest and that there may be differences in the location of cell cycle arrest between different cell types.

MDA-MB-231 and 4 T1 cells were treated with different concentrations of STC2 (0.25, 0.5, and 1 *μ*M) for 6 h, and the expression of cycle-related proteins in MDA-MB-231 and 4 T1 cells was detected by western blot [17]. The results showed that STC2 downregulated the expression of cyclin B1 and phosphorylated CDK1 in MDA-MB-231 cells. The expression levels of cyclin A2 and the corresponding CDK2 binding protein were downregulated in 4 T1 cells (Figures 3(c) and 3(d)). Combined with the results of the cell cycle experiment, this indicates that the sea cucumber saponin STC2 can inhibit the smooth transformation of the cell cycle from the G2 phase to the M phase by downregulating the protein expression level of cyclin B1 and the phosphorylation level of CDK1 in MDA-MB-231 cells, thus arresting the cell cycle in the G2/M phase. In addition, STC2 can inhibit the smooth transformation of the cell cycle from the S phase to the G2 phase in 4 T1 cells by downregulating the protein expression levels of cyclin A2 and CDK2, thus arresting the cell cycle in the S phase.

### 3.4. STC2 Induces DNA Damage of Breast Cancer Cells

**Figure 4:**
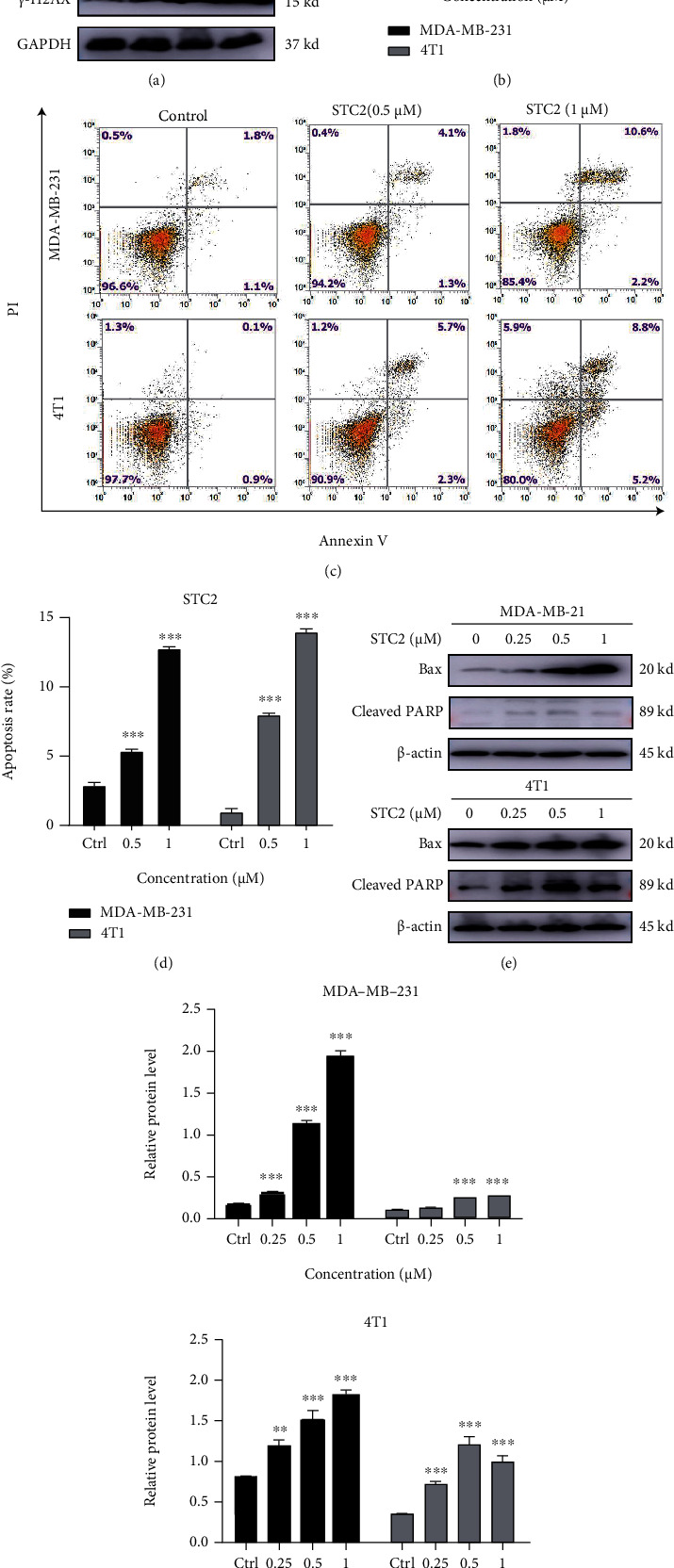
Sea cucumber saponins induced DNA damage and apoptosis of triple-negative breast cancer (TNBC) cells. (a) Western blot was used to detect the phosphorylation of H2AX in both cells. (c) MDA-MB-231 and 4 T1 cells were treated with 0.5 and 1 *μ*M stichloroside C2 (STC2) for 24 h. The cells were digested and collected. The Annexin V-FITC apoptosis kit was used to treat the cells. The average fluorescence intensity of Annexin V/PI was determined by flow cytometry. (e) Bax and cleaved PARP protein expression levels were detected by western blot. (b, d, and f) Prism 8.0 was used for the statistical analysis of experimental data, which were expressed as the mean ± SEM of three independent experiments. ∗*P* < 0.05, ∗∗*P* < 0.01, ∗∗∗*P* < 0.001, compared to the control group.

DNA double-strand breaks are among the most serious types of DNA damage [18]. *γ*-H2AX, formed after the phosphorylation of the histone H2AX, is closely associated with—and can be used as a marker of—DNA double-strand breaks [19]. The human TNBC cell line MDA-MB-231 and the mouse breast cancer cell line 4 T1 were treated with a series of different concentrations (0.25, 0.5, and 1 *μ*M) of STC2 for 24 h. The treated cells were collected, and total protein was extracted. Western blotting was used to detect the phosphorylation levels of *γ*-H2AX and H2AX. As shown in Figures 4(a) and 4(b), the expression levels of *γ*-H2AX in both STC2-treated cells showed a significant upregulation trend (*P* < 0.001). Therefore, it can be concluded that STC2 can induce DNA damage in the two types of breast cancer cells.

### 3.5. STC2 Induces Apoptosis of Breast Cancer Cells

Apoptosis is closely related to DNA damage [20, 21]. When DNA damage occurs in tumour cells, the DNA damage response system of the cell is rapidly activated to repair the damaged DNA [22]. If the repair fails due to the failure of the DNA damage response system owing to certain factors, the cell will enter the death process, leading to apoptosis. To study the effect of sea cucumber saponins on TNBC cell apoptosis, the apoptosis of breast cancer cells treated with sea cucumber saponins was detected by flow cytometry. MDA-MB-231 and 4 T1 cells were treated with different concentrations of STC2 (0.5, 1 *μ*M) for 24 h, digested, and collected. The Annexin V-FITC apoptosis kit was used to treat cells. The average fluorescence intensity of Annexin V/PI was determined by flow cytometry. The results showed that STC2 induced apoptosis in both breast cancer cell types in a dose-dependent manner (Figures 4(c) and 4(d)). In combination with previous experimental results, this result suggested that the DNA damage caused by sea cucumber saponins on breast cancer cells might trigger apoptosis in breast cancer cells.

MDA-MB-231 and 4 T1 cells were treated with different concentrations of STC2 (0.25, 0.5, and 1 *μ*M) for 6 h to detect the expression of apoptosis-related proteins in breast cancer cells. Bax and cleaved PARP protein expression levels were detected using western blotting. The results showed that after STC2 was used to treat the two breast cancer cell lines, Bax protein expression levels were significantly upregulated, and cleaved PARP protein expression levels were also upregulated to varying degrees (Figures 4(e) and 4(f)). The expression of cleaved PARP was significantly upregulated in 4 T1 cells. These results indicated that the sea cucumber saponin monomer STC2 can induce the apoptosis of breast tumour cells by regulating the expression levels of apoptosis-related proteins. According to the *γ*-H2AX protein expression level before binding, the sea cucumber saponin monomer STC2 may promote the apoptosis of breast cancer cells by stimulating the regulation of DNA damage response-related proteins.

### 3.6. STC2 Inhibits EMT in TNBC Cells

**Figure 5:**
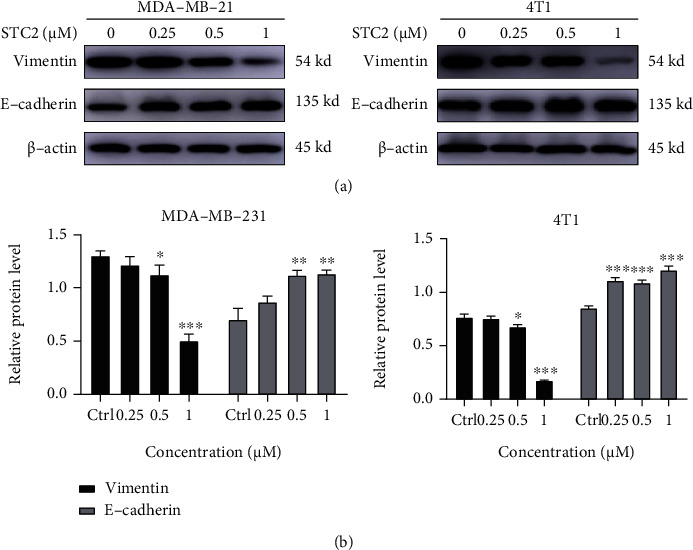
Stichloroside C2 (STC2) inhibited epithelial–mesenchymal transition (EMT) in triple-negative breast cancer (TNBC) cells. (a) MDA-MB-231 and 4 T1 cells were treated with different concentrations of STC2 (0.25, 0.5, and 1 *μ*M) for 6 h, and the protein expression levels of E-cadherin and vimentin were detected by western blot. (b) Data are presented as the mean ± SEM of three independent experiments, ∗*P* < 0.05, ∗∗*P* < 0.01, ∗∗∗*P* < 0.001, compared to the control group.

The invasion and metastasis of TNBC are closely related to the occurrence of EMT [23]. EMT pathogenesis is regulated by a series of related transcription factors, among which E-cadherin is a marker of the epithelial phenotype and vimentin is a marker of the interstitial phenotype [24]. EMT can upregulate the expression of vimentin and downregulate the expression of the epithelial cell biomarker E-cadherin. To verify the effect of STC2 on the EMT in TNBC cells, MDA-MB-231 and 4 T1 cells were treated with different concentrations of STC2 (0.25, 0.5, and 1 *μ*M) for 6 h. The protein expression levels of E-cadherin and vimentin were detected using western blotting. The results showed that STC2 significantly promoted the protein expression of the epithelial biomarker E-cadherin and inhibited the protein expression of the interstitial phenotypic marker vimentin (Figure 5). These results indicate that STC2 can inhibit the occurrence of EMT in TNBC cells.

### 3.7. Effects of STC2 on the Phosphorylation Level of MAPK Pathway-Related Proteins in TNBC Cells

The MAPK signalling pathway is a key pathway for cell apoptosis and the induction of many malignant tumours [25]. It is also an important pathway for the occurrence of EMT [26]. Therefore, to further explore the mechanism by which STC2 induces TNBC cell apoptosis and inhibits EMT, western blotting was performed to detect the expression of related proteins.

**Figure 6:**
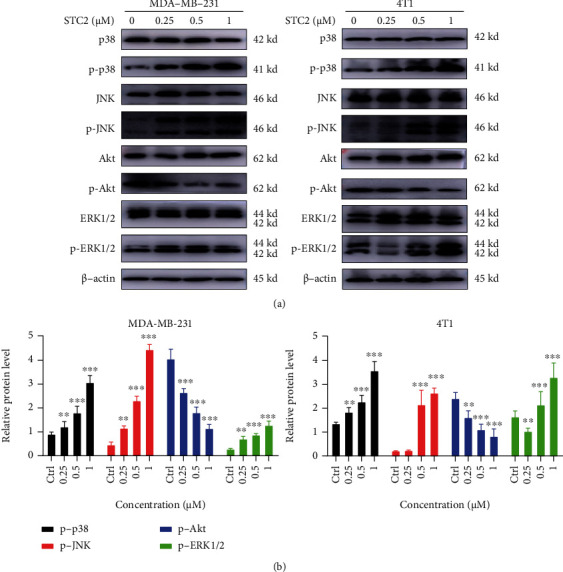
Effect of stichloroside C2 (STC2) on MAPK pathway-related protein expression in triple-negative breast cancer (TNBC) cells. (a) MDA-MB-231 and 4 T1 cells were treated with different concentrations of STC2 (0.25, 0.5, and 1 *μ*M) for 6 h. Western blotting was used to detect the protein expression and phosphorylation levels of p38, JNK, ERK1/2, and Akt, the main subfamilies of the MAPK signalling pathway family. (b) Data are presented as the mean ± SEM of three independent experiments ∗∗*P* < 0.01, ∗∗∗*P* < 0.001, compared to the control group.

MDA-MB-231 and 4 T1 cells were treated with different concentrations of STC2 (0.25, 0.5, and 1 *μ*M) for 6 h. Western blotting was used to detect the protein expression and phosphorylation levels of p38, JNK, ERK1/2, and Akt, the main subfamilies of the MAPK signalling pathway family. The results showed that STC2 could significantly upregulate the phosphorylation levels of P38, JNK, and ERK1/2 and significantly downregulate the phosphorylation level of Akt in TNBC cells (Figure 6). The results showed that STC2 inhibited the proliferation of breast cancer cells and promoted the apoptosis of TNBC cells by inhibiting the phosphorylation of Akt and reducing its phosphorylation level in TNBC cells, thereby blocking the Akt-related signalling pathway. STC2 promoted the phosphorylation of P38, JNK, and ERK1/2 and increased their phosphorylation levels in TNBC cells, suggesting that STC2 can activate the relevant pathways of MAPK, a classical signalling pathway, to induce the apoptosis of TNBC cells and inhibit the occurrence of EMT.

## 4. Discussion

Sea cucumbers have been considered nutrient-rich foods with various biological activities for centuries. They produce highly active substances, especially saponins, which are the main secondary metabolites of sea cucumbers and are the basis of their chemical defence [27]. Saponins contain triterpenoids or steroids, which have a variety of biological characteristics, including antitumour and hypolipidaemic activity, improvement of nonalcoholic fatty liver, inhibition of fat accumulation, antihyperuricemia, promotion of bone marrow haematopoiesis, and antihypertension [28]. We adopted advanced natural compound extraction and separation methods to successfully obtain four saponins from *Thelenota ananas*, which were isolated from monomers, and identified their chemical structures through the high-resolution mass spectrometry and nuclear magnetic resonance analysis. STC2, with its strong antitumour activity and low cytotoxicity to normal cells, was selected for further experiments.

CDKs and cyclins are usually the first targets to be inhibited when abnormal intracellular events, such as abnormal pressure, telomerase dysfunction, and DNA damage, occur because of their important roles in the whole cell cycle regulation system. When DNA damage occurs in cells, the DNA repair system will actively block the cycle by activating the cell cycle checkpoint (checkpoint), which is mainly responsible for the system run-time monitoring feedback signals, and cell cycle regulation in the system when an exception occurs in a timely manner according to the instructions to block the cell cycle signal process. Therefore, the cell cycle checkpoint regulation mechanism is closely related to the occurrence and development of tumours [29]. By detecting the effects of STC2 and the expression levels of post-TNBC cell cycle-related proteins, we found that STC2-targeted MDA-MB-231 and 4 T1 cells can participate in key sites of cyclin B/CDK1 and cyclin A/CDK2 cell cycle transformation. Thus, the cell cycle was arrested in the G2/M and S phases, which provided a preliminary verification of the molecular mechanism of the results obtained in the second part. By investigating the effects of STC2 on the expression levels of apoptosis-related proteins Bax and PARP, combined with the conclusions of the previous section, STC2-induced apoptosis by DNA damage was further confirmed, suggesting that STC2 promotes the apoptosis of breast cancer cells by stimulating and regulating the DNA-damage-related proteins.

MAPK is a classic and significant signalling pathway, and its family includes many subfamilies, including the P38-MAPK, ERK1/2, and JNK pathways, which also constitute multiple signalling pathways [30]. The MAPK signalling pathway is a common pathway of extracellular signal stimulation that induces a nuclear response and is an intracellular signalling molecule mediating EMT. Several signalling molecules mediate EMT via the MAPK signalling pathway. After external stimulation, MAPK is doubly phosphorylated, and cytoplasmic signals are transferred to the nucleus by MAPK and play corresponding roles. Studies have shown that MAPK signalling is associated with the development and progression of TNBC [31]. In this study, by detecting the expression and phosphorylation levels of its main subfamily proteins, we found that STC2 inhibited the proliferation of breast cancer cells and promoted the apoptosis of TNBC cells by inhibiting the phosphorylation of Akt and blocking the Akt-related signalling pathway. STC2 promoted the phosphorylation of P38, JNK, and ERK1/2 and increased the phosphorylation levels of P38, JNK, and ERK1/2 in TNBC cells, suggesting that it can activate the relevant pathways of MAPK, a classical signalling pathway, to induce apoptosis in TNBC cells and inhibit the occurrence of EMT. These results provide a direction for further exploration of the antitumour mechanism of STC2 in the future.

**Figure 7:**
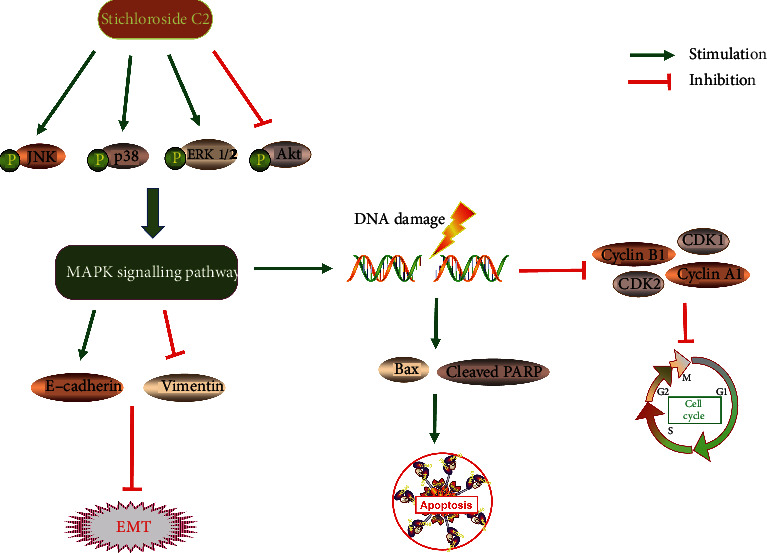
Schematic diagram of STC2 inhibiting EMT and inducing apoptosis of TNBC cells through MAPK signalling pathway.

In conclusion, we identified a potential anti-TNBC hit compound, STC2, by combining biological and chemical techniques. We found that STC2 significantly inhibited the proliferation of TNBC cells, induced cell cycle arrest and apoptosis in TNBC cells, and inhibited the occurrence of EMT; these effects are closely related to the Akt and MAPK signalling pathways (Figure 7). These results provide convincing evidence for the future preclinical use of STC2 as a promising compound for the treatment of human TNBC. Further exploration of the antitumour effect of STC2 on the MAPK signalling pathway and action target studies is in progress.

## Data Availability

The data used to support the findings of this study are available from the corresponding author upon reasonable request.

## Supplementary Materials

Supplementary Materials The structures of the isolated compounds were identified by comparing their MS and NMR data with those of the literature values [1–5]. Stichloroside C2 (STC2): white powder. ^1^H and ^13^C NMR data (Pyridine-d_5_) see Table S1 and S2, HRESIMS *m/z* 1436.6815 [M-H]^−^ (C_68_H_107_O_32_, calcd. 1436.6824). Stichloroside B1 (STB1): white powder. ^1^H and ^13^C NMR data (Pyridine-d_5_) see Table S1 and S2, HRESIMS *m/z* 1454.6908 [M-H]^−^ (C_68_H_109_O_33_, calcd. 1454.6929). Stichoposide C1 (STC1): white powder. ^1^H and ^13^C NMR data (Pyridine-d_5_) see Table S1 and S2, HRESIMS *m/z* 1438.6964 [M-H]^−^ (C_68_H_109_O_32_, calcd. 1438.6980). Stichoposide A (STA): light yellow powder. ^1^H and ^13^C NMR data (Pyridine-d_5_) see Table S1 and S2, HRESIMS *m/z* 792.4693 [M-H]^−^ (C_43_H_67_O_13_, calcd. 792.4660).
