# Supplementary material for: Marine-Derived Stichloroside C2 Inhibits Epithelial–Mesenchymal Transition and Induces Apoptosis through the Mitogen-Activated Protein Kinase Signalling Pathway in Triple-Negative Breast Cancer Cells
Source: J Oncol. 2022 May 14;2022:6449984. doi: 10.1155/2022/6449984 (PMC9124082; doi:10.1155/2022/6449984)
Supplement: Supplementary Materials — The structures of the isolated compounds were identified by comparing their MS and NMR data with those of the literature values [1–5]. Stichloroside C2 (STC2): white powder. 1H and 13C NMR data (Pyridine-d5) see Table S1 and S2, HRESIMS m/z 1436.6815 [M-H]− (C68H107O32, calcd. 1436.6824). Stichloroside B1 (STB1): white powder. 1H and 13C NMR data (Pyridine-d5) see Table S1 and S2, HRESIMS m/z 1454.6908 [M-H]− (C68H109O33, calcd. 1454.6929). Stichoposide C1 (STC1): white powder. 1H and 13C NMR data (Pyridine-d5) see Table S1 and S2, HRESIMS m/z 1438.6964 [M-H]− (C68H109O32, calcd. 1438.6980). Stichoposide A (STA): light yellow powder. 1H and 13C NMR data (Pyridine-d5) see Table S1 and S2, HRESIMS m/z 792.4693 [M-H]− (C43H67O13, calcd. 792.4660). [file 6449984.f1.pdf]

Supplementary materials

Marine-derived Stichloroside C2 Inhibits Epithelial–mesenchymal Transition and Induces Apoptosis through the Mitogen-activated Protein Kinase Signalling Pathway in Triple-negative Breast Cancer Cells

Chuang Cui, Chen-Huan Ding, Fang-Fang Liu, Jing-Rong Lu, Shi-Yun Zheng, Hou-Wen Lin, Wei-Kang Zhu*, Fan Yang*, He Li*

The structures of the isolated compounds were identified by comparing their MS and NMR data with those of the literature values [1-5].

Stichloroside C2 (STC2): white powder. ^{1}H and ^{13}C NMR data (Pyridine-d_5) see Table S1 and S2, HRESIMS *m/z* 1436.6815 [M-H]$^-$ ($C_{68}H_{107}O_{32}$, calcd. 1436.6824).

Stichloroside B1 (STB1): white powder. ^{1}H and ^{13}C NMR data (Pyridine-d_5) see Table S1 and S2, HRESIMS *m/z* 1454.6908 [M-H]$^-$ ($C_{68}H_{109}O_{33}$, calcd. 1454.6929).

Stichoposide C1 (STC1): white powder. ^{1}H and ^{13}C NMR data (Pyridine-d_5) see Table S1 and S2, HRESIMS *m/z* 1438.6964 [M-H]$^-$ ($C_{68}H_{109}O_{32}$, calcd. 1438.6980).

Stichoposide A (STA): light yellow powder. ^{1}H and ^{13}C NMR data (Pyridine-d_5) see Table S1 and S2, HRESIMS *m/z* 792.4693 [M-H]$^-$ ($C_{43}H_{67}O_{13}$, calcd. 792.4660).

Table S1 NMR data of aglycones of sea cucumber saponins (Pyridine-d_5, 600 MHz)

C	**STC2**		**STB1**		**STC1**		**STA**	
	^{13}C(mult.)	^{1}H(mult.)	^{13}C(mult.)	^{1}H(mult.)	^{13}C(mult.)	^{1}H(mult)	^{13}C(mult.)	^{1}H(mult)
1	36.1	1.44, m	36.1	1.46, m	35.8	1.43, m	37.5	1.40, m
2	26.9	1.89, m/2.09, m	26.9	1.92, m/2.11, m	27.2	1.90, m/2.08, m	25.6	1.85, m/2.07, m
3	88.9	3.25, m	88.7	3.27, m	89.3	3.24, m	92.6	3.04, m
4	39.4	-	39.3	-	39.8	-	39.2	-
5	47.8	0.95, m	47.8	0.97, m	47.6	0.99, m	50.1	1.07, m
6	23.1	1.94, m	23.1	1.96, m	23.4	2.02, m	23.5	1.94, m
7	119.8	5.63, br s	119.8	5.65, br s	120.3	5.65, br s	123.0	5.27, br s
8	146.4	-	146.4	-	146.9	-	146.8	-
9	47.2	3.42, m	47.2	3.44, m	47.6	3.42, m	47.3	3.66, m
10	35.4	-	35.4	-	36.5	-	35.7	-
11	22.9	1.44, m/1.72, m	22.8	1.46, m/1.73, m	22.5	1.44, m/1.72, m	21.0	1.45, m/1.78, m
12	30.0	1.90, m	30.1	1.87, m/1.94, m	30.1	1.89, m	31.4	1.67, m
13	58.2	-	58.3	-	58.7	-	59.4	-
14	51.0	-	51.1	-	51.6	-	51.5	-
15	34.0	1.62, m/1.74, m	34.1	1.63, m/1.76, m	34.5	1.62, m/1.74, m	33.8	1.56, m/1.81, m
16	24.5	1.89, m/2.05, m	24.6	1.91, m/2.07, m	24.8	1.88, m	25.1	1.78, m

17	53.8	2.30, m	54.0	2.32, m	54.4	2.29, m	54.5	2.19, m
18	179.6	-	179.7	-	180.1	-	180.2	-
19	23.8	1.18, s	23.9	1.20, s	23.6	1.20, s	14.8	1.12, s
20	83.3	-	83.0	-	83.8	-	85.9	-
21	26.7	1.49, s	26.8	1.51, s	27.2	1.48, s	28.4	1.39, s
22	43.8	1.86, m/2.16, m	43.9	1.88, m/2.17, m	44.3	1.85, m/2.14, m	39.4	1.43, m
23	67.7	5.41, m	68.1	5.43, m	68.5	5.40, m	66.7	5.32, m
24	45.1	1.25, m/1.55, m	45.2	1.27, m/1.58, m	45.6	1.23, m/1.54, m	39.9	1.19, m
25	141.1	-	24.4	1.58, m	24.5	1.54, m/1.89, m	28.1	1.62, m
26	23.1	1.94, m	23.0	0.89, d	23.2	0.85, d	23.2	0.91, d
27	114.1	4.90, 4.88, br s	21.9	0.94, d	22.3	0.90, d	23.2	0.91, d
28	28.5	1.21, s	28.6	1.23, s	28.9	1.26, s	23.8	0.89, s
29	17.1	1.07, s	17.2	1.11, s	17.6	1.11, s	23.8	0.89, s
30	30.7	1.03, s	30.8	1.06, s	30.5	1.04, s	32.3	1.01, s
OAc	170.3	-	170.6	-	171.0	-	170.2	-
OAc	20.9	2.13, s	21.2	2.15, s	21.6	2.12, s	21.0	2.02, s

Table S2 NMR data of polysaccharide groups of sea cucumber saponins (Pyridine-d_5, 600 MHz)

C	**STC2**		**STB1**		**STC1**		**STA**	
	^{13}C(mult.)	^{1}H(mult)	^{13}C(mult.)	^{1}H(mult)	^{13}C(mult.)	^{1}H(mult.)	^{13}C(mult.)	^{1}H(mult.)
Xyl[1]								
1	105.2	4.74, d	105.1	4.76, d	105.4	4.80, d	111.7	5.60, d
2	82.9	4.09	83.4	4.11	83.5	3.99	83.6	3.60
3	75.5	4.20	75.5	4.20	75.7	4.17	73.7	3.90
4	77.2	4.24	77.4	4.25	77.6	4.23	70.8	3.70
5	63.8	3.62/4.39	63.8	3.65/4.40	64.3	3.60/4.36	66.5	3.63/3.88
Glu[1]							*Qui*	
1	105.3	5.22, d	105.5	5.24, d	105.6	5.24, d	110.1	5.40, d
2	76.2	4.03	76.4	4.04	76.0	3.98	74.2	3.70
3	87.6	4.24	86.5	3.81	87.9	4.26	73.7	3.60

4	70.4	4.25	70.1	4.37	70.9	4.33	72.4	3.70
5	77.9	3.92	78.2	3.95	78.5	3.91	74.1	3.90
6	61.8	4.23/4.43	62.0	4.25/4.45	62.3	4.20/4.42	18.7	1.20, d
Xyl[2]								
1	104.9	5.08, d	104.7	5.10, d	105.5	5.10, d		
2	73.3	3.97	74.9	3.98	75.3	3.95		
3	87.6	4.19	87.9	4.20	87.6	4.14		
4	68.8	4.01	68.9	4.02	69.3	4.00		
5	66.3	3.54/4.15	66.4	3.56/4.17	66.7	3.58/4.16		
Me-Glu[1]								
1	105.3	5.22, d	105.5	5.24, d	105.6	5.24, d		
2	74.9	4.96	74.9	4.98	75.3	4.95		
3	87.7	3.69	87.8	3.70	88.2	3.66		
4	70.3	4.10	70.4	4.12	70.7	4.08		
5	78.1	3.92	78.2	3.95	78.5	3.91		
6	61.9	4.23/4.43	62.0	4.25/4.45	62.4	4.20/4.42		
3-OMe	60.5	3.84	60.6	3.85	60.9	3.81		
Qui			*Glu*[2]					
1	102.5	4.96, d	102.7	4.98, d	103.0	4.94, d		
2	73.3	3.97	74.9	3.98	73.3	3.95		
3	77.8	4.04	77.4	4.05	77.6	3.96		
4	88.8	4.00	88.9	4.02	89.0	4.08		
5	78.1	3.92	78.2	3.95	78.5	3.91		
6	18.9	1.69, d	62.0	4.25/4.45	18.8	1.72, d		
Me-Glu[2]								
1	105.3	5.22, d	105.5	5.24, d	105.6	5.24, d		
2	74.9	3.96	74.9	3.98	75.3	3.95		
3	87.7	3.69	87.9	3.70	88.2	3.66		
4	70.3	4.11	70.4	4.12	70.7	4.08		
5	78.1	3.96	78.2	3.95	78.5	3.91		
6	61.9	4.23/4.43	62.0	4.25/4.45	62.4	4.20/4.42		
3-OMe	60.6	3.84	60.6	3.85	60.9	3.81		

In order to screen out suitable compounds for further research. Four cell lines (MDA-MB-231, 4T1, MCF-7, and IOSE-80) were tested by CCK-8 assay with different concentrations of STC2, STB1, STC1 and STA. The results showed that the four compounds had significant inhibitory effects on the activity of breast cancer cells

(Figure S1, Table S3). However, they also have a strong killing effect on normal epithelial cells, among which STC2 is relatively less lethal to normal cells. Therefore, we chose STC2 for further in vitro experiments.

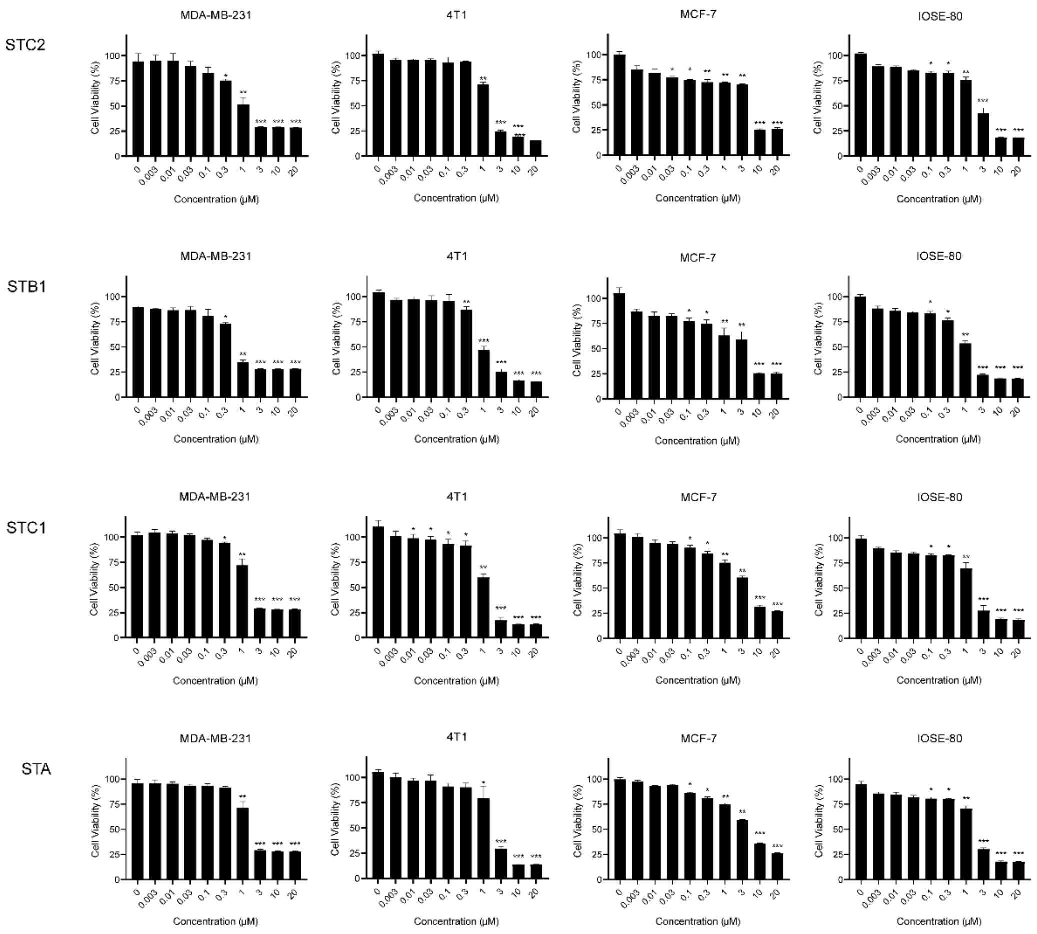

Figure S1 Inhibitory effects of four sea cucumber saponins on different tumor cells. Three breast cancer cell lines including human breast cancer cell line (MCF-7), human TNBC cell line (MDA-MB-231), mouse breast cancer cell line (4T1) and a normal epithelial cell line (IOSE-80) were selected and treated with a series of different concentrations of STC2, STB1, STC1 and STA respectively After treatment for 24 h, the cell viability was detected by CCK-8 method.*P＜0.05,**P＜0.01,***P＜0.001,compared with control group.

Table S3 IC_{50} values of four compounds to several cell lines (μM)

	STC2	STB1	STC1	STA
MDA-MB-231	1.46±0.15	1.94±0.19	2.49±0.90	2.42±0.86
4T1	1.84±0.46	1.20±0.77	1.29±0.49	2.04±0.91
MCF-7	3.38±0.91	2.42±0.34	4.39±0.87	4.38±1.05
IOSE-80	2.19±0.65	1.00±0.13	1.55±0.43	1.48±0.39

References:

[1] Findlay J A, Daljeet A. Frondogenin, a New Aglycone From the Sea Cucumber Cucumaria Frondosa. J Nat Prod, 1984, 2: 320-324

[2] Cuong N X, Vien L T, Hoang L et al. Cytotoxic Triterpene Diglycosides From the Sea Cucumber Stichopus Horrens. Bioorg Med Chem Lett, 2017, 13: 2939-2942

[3] Thao N P, Luyen B T, Le Vien T et al. Triterpene Saponins From the Sea Cucumber Stichopus Chloronotus. Nat Prod Commun, 2014, 5: 615-618

[4] Vien L T, Hoang L, Hanh T et al. Triterpene Tetraglycosides From the Sea Cucumber Stichopus Horrens. Nat Prod Res, 2018, 9: 1039-1043

[5] Yun S H, Park E S, Shin S W et al. Stichoposide C Induces Apoptosis through the Generation of Ceramide in Leukemia and Colorectal Cancer Cells and Shows in Vivo Antitumor Activity. Clin Cancer Res, 2012, 21: 5934-5948
